# Exogenous silicon promotes cadmium (Cd) accumulation in *Sedum alfredii* Hance by enhancing Cd uptake and alleviating Cd toxicity

**DOI:** 10.3389/fpls.2023.1134370

**Published:** 2023-02-21

**Authors:** Ying Hu, Xueqi Zhou, An Shi, Yanshuang Yu, Christopher Rensing, Taoxiang Zhang, Shihe Xing, Wenhao Yang

**Affiliations:** ^1^ Key Laboratory of Soil Ecosystem Health and Regulation of Fujian Provincial University, College of Resources and Environment, Fujian Agriculture and Forestry University, Fuzhou, China; ^2^ College of Forestry, Fujian Agriculture and Forestry University, Fuzhou, China

**Keywords:** silicon, antioxidant enzyme activity, cell wall, organic acids, hyperaccumulator

## Abstract

Soil Cadmium (Cd) pollution has become a serious environmental problem. Silicon (Si) plays key roles in alleviating Cd toxicity in plants. However, the effects of Si on mitigation of Cd toxicity and accumulation of Cd by hyperaccumulators are largely unknown. This study was conducted to investigate the effect of Si on Cd accumulation and the physiological characteristics of Cd hyperaccumulator *Sedum alfredii Hance* under Cd stress. Results showed that, exogenous Si application promoted the biomass, Cd translocation and concentration of *S. alfredii*, with an increased rate of 21.74-52.17% for shoot biomass, and 412.39-621.00% for Cd accumulation. Moreover, Si alleviated Cd toxicity by: (i) increasing chlorophyll contents, (ii) improving antioxidant enzymes, (iii) enhancing cell wall components (lignin, cellulose, hemicellulose and pectin), (iv) raising the secretion of organic acids (oxalic acid, tartaric acid and L-malic acid). The RT-PCR analysis of genes that involved in Cd detoxification showed that the expression of *SaNramp3*, *SaNramp6*, *SaHMA2* and *SaHMA4* in roots were significantly decreased by 11.46-28.23%, 6.61-65.19%, 38.47-80.87%, 44.80-69.85% and 33.96-71.70% in the Si treatments, while Si significantly increased the expression of *SaCAD*. This study expanded understanding on the role of Si in phytoextraction and provided a feasible strategy for assisting phytoextraction Cd by *S. alfredii*. In summary, Si facilitated the Cd phytoextraction of *S. alfredii* by promoting plant growth and enhancing the resistance of plants to Cd.

## 1 Introduction

Soil heavy metal contamination caused by various anthropogenic activities has become a serious global environmental problem. Among the heavy metals, cadmium (Cd) is regarded as no known biological functions but highly harmful to the organisms (Gul et al., 2021). Due to the persistence of Cd in soil, Cd has been shown to strongly affect the growth and biomass development and cause plant poisoning (Gallego et al., 2012). In addition, Cd is easily absorbed by grain due to the high mobility of Cd, which will eventually affect animal and human health through the food chain (Żukowska and Biziuk, 2010; Xie et al., 2020). Therefore, effective methods are needed to eliminate Cd contaminated soil for assuring the environmental health and food security.

In the past few decades, various chemical and physical techniques have been developed for the remediation of Cd-contaminated soils. However, these methods usually display high costs, disturbance of soils and have environmental risks (Gul et al., 2021; Gavrilescu, 2022). Phytoextraction is currently considered an eco-friendly and low cost approach to remove heavy metals from soil. This technology based on the capacity of plants to uptake heavy metals from soil and then accumulate them in aboveground parts of the plant, without adverse effects on soil structure, fertility and biological activity (Kurade et al., 2021; Gavrilescu, 2022). The success of phytoextraction depends largely on the uptake and accumulation of metals by hyperaccumulators. However, their characteristics of slow growth and small biomass under metal stress severely affect the phytoextraction efficiency, which has become an obstacle for the large-scale application of phytoextraction (Feng et al., 2018). Therefore, from the point of view of increasing plant biomass to alleviate the toxicity of Cd for plants, a strategy for improving the removal efficiency may provide an effective technology for solving this setback of phytoextraction.

Silicon (Si), the second most abundant element in the earth crust after oxygen, is an important element for plants (Emamverdian et al., 2018; Bhat et al., 2019), which playing an important role in the survival of plants under heavy metal stress. After being absorbed by the plants, Si was able to reduce the harmful effects of heavy metal toxicity through various mechanisms, including stimulating the antioxidant defense system (Huang et al., 2019; El-Saadony et al., 2021), reducing metal uptake and transport (Gao et al., 2018; Chen et al., 2019; Gheshlaghpour et al., 2021), complexing (Yamaji et al., 2008; Keller et al., 2014; Bhat et al., 2019), compartmentalization (Richmond and Sussman, 2003; Gu et al., 2011) and altering the components of the cell wall or forming a bilayer cell structure (Fan et al., 2016). In addition, Si was also able to reduce the metal phyto-toxicity at the molecular level by elevating transcription of genes encoding metal transporters and phyto-chelatine (Vaculík et al., 2020). In general, the current research have demonstrated that Si addition is an effective strategy to alleviate toxicity caused by heavy metals. However, most of these studies only focused on the effects of Si on non-hyperaccumulating plants. To the best of our knowledge, there is also a research gap in the effects of Si on the mitigation of Cd toxicity as well as the uptake and accumulation of Cd by hyperaccumulators.

*Sedum alfredii Hance*, originally discovered in an old mining site in Southeast China, has been reported to be a naturally occurring Cd hyperaccumulator with a great potential in Cd phytoextraction (Yang et al., 2019). And *S. alfredii* has been used as a model hyperaccumulator to investigate Cd phytoextraction owing to its strong ability of Cd uptake and accumulation (Xu et al., 2023). To date, synergistic effects of Si on mitigation of Cd toxicity and accumulation of Cd by hyperaccumulators have not been reported. Hence, in this study, the physiological response of *S. alfredii* to exogenous Si application under Cd stress was comprehensively studied by a hydroponic experiment. The objectives were to: 1) determine the influence of Si on growth and Cd accumulation by *S. alfredii*; 2) investigate the effects of Si on the mitigation of plant Cd stress by measuring the root exudation of organic acids, cell wall constituents, level of lipid peroxidation, chlorophyll content, and antioxidant enzymes; 3) explore the response to Cd uptake and subsequent expression of genes encoding specific transporter (*SaNramp3*, *SaNramp6*, *SaHMA2*, *SaHMA3* and *SaHMA4*) and cell wall synthetase (*SaCAD*) under Cd stress. The results of this study will improve our understanding of role of Si in phytoextraction of Cd by hyperaccumulators.

## 2 Materials and method

### 2.1 Plant material and experimental design

The seedlings of *S. alfredii* were obtained from a mine area in Zhejiang Province, China. The plants were grown in uncontaminated soil for more than 3 generations to eliminate the influence of the previously accumulated heavy metals in the plants. *S. alfredii* plants with consistent growth conditions were pre-cultured in Hoagland nutrient solution (pH 5.8), which contained KH_2_PO_4_ 100μM, Fe-EDTA 20μM, CuSO4·5H_2_O 0.2μM, Ca(NO_3_)_2_·4H_2_O 2000μM, ZnSO_4_·7H_2_O 0.5μM, (NH_4_)6Mo_7_O_24_ 0.01μM, H_3_BO_3_ 10μM, MnSO_4_·H_2_O 0.5μM, MgSO_4_·7H_2_O 500μM, K_2_SO_4_ 700μM, KCl 100μM. *S. alfredii* was grown in a greenhouse at 26/20°C with a day/night cycle of 16h/8h, and the humidity was kept at about 70%. The nutrient solution was replaced every 3 days and continuously aerated. After 21 days of pre-culture, healthy seedlings (the seedling height is 6~7cm and the root length is 3~4cm) were subsequently chosen for the later hydroponic experiment.

The hydroponic experiment was conducted in a controlled greenhouse. During the experiment, *S. alfredii* was grown in a greenhouse at 26/20°C with a day/night cycle of 16h/8h, and the humidity was kept at about 70%. Four uniform plants were selected and transferred to each pot (diameter 15.5 cm and height 10 cm). All treatments contained 0.15 mM Cd, which was supplied as CdCl_2_. Sodium metasilicate (Na_2_SiO_3_·9H_2_O, SiO_2_ 21%) was applied as a source of Si. Na_2_SiO_3_ at different concentrations (0, 0.5, 1.0, 1.5 and 2.0 mM) were dissolved in nutrient solution, marked CK, T1, T2, T3 and T4, respectively. The HCl was used to neutralize the alkalinity of Na_2_SiO_3_, and the difference of Na^+^ and Cl^-^ among each treatment were made up with NaCl. Each treatment was set up with three replications with a total of 15 pots. The nutrient solution was continuously aerated with an aquarium air pump and renewed every 3 days, and the pH value was adjusted to 5.8.

### 2.2 Plant harvesting and Cd/Si concentrations analysis

After 28 d growth, plants were harvested for analysis of morphological, physiological and molecular characteristics. After harvest, *S. alfredii* plants were fully washed with deionized water and divided into shoot and root. The root length, plant height and fresh weight of plants were measured by ruler and electronic balance, respectively. The plant samples were dried to constant weight and then recorded dry weight. The dried plant material was then ground to a fine powder for the determination of metal concentrations. To measure Cd concentrations of plants, the powdered plant material (0.1g) was digested with a mixture of HNO_3_ and HClO_4_ (5:1) on an electric heating plate. The concentration of Cd in the digested solution was determined by using ICP-MS (PerkinElmer NexION 300X). A standard plant material (GSB–11) was used throughout all the digestions and ICP analysis processes as the quality control. The translocation factors (TF) for Cd were calculated according to the following formula:


TF= Metal concentration in plant shoots/Metal concentration in plant roots

The Si content was measured by Si molybdenum blue colorimetry (Yang, 1995). Briefly, the oven-dried root and shoot samples (0.1g) were grinded with the help of a mortar. The samples were then placed on a high-temperature electric resistance furnace and heated at 720 ℃ for 10 min. Later on, the samples were mixed with 0.3 mol∙L^-1^ H_2_SO_4_, 5% ammonium molybdate, 5% oxalic acid, and 0.5% ascorbic acid. Then the Si content was photometrically measured at a wavelength of 700 nm.

### 2.3 Analysis of antioxidant enzyme activities, lipid peroxidation and chlorophyll contents

To evaluate the antioxidant enzyme activities of plants, fresh leaves (0.2g) were reacted with potassium phosphate buffer (pH 7.8). The homogenate was centrifuged at 8000×g for 10 minutes was stored at 4 ℃ for the enzyme activity determination. The SOD (superoxide dismutase) was assayed in 200 μl of reaction mixture containing 10 μl enzyme extract, 160 μl phosphate buffer (pH 7.8) and 30 μl WST-8. Then the samples were measured by using a spectrophotometer at 450nm wavelength. Finally, the enzyme activity was calculated using SOD activity determination kit (Sino Best Biological Technology Co., Ltd, Shanghai, China). The CAT (catalase) was determined according to a previous method (Aebi, 1984). The reaction mixture (3mL) was comprised of 100μl enzyme extract, 100 μl H_2_O_2_ and 2.8mL phosphate buffer with EDTA (pH 7.0). The absorbance of the reaction mixture at 240nm decreases with the reaction time, and the CAT activity can be calculated according to the change rate of absorbance. The POD (peroxidase) was analyzed based on a previous method (Farooq et al., 2013). The assay mixture (245μl) was consisted of 5μl enzyme extract, 120μl guaiacol, 30μl H_2_O_2_ (300 mM), 30μl phosphate buffer with EDTA (pH 7.0) and 60μl distilled water. The absorbance of guaiacol oxidation products was determined at 470nm wavelength.

The level of lipid peroxidation in the leaf tissue was measured in terms of malondialdehyde (MDA, a product of lipid peroxidation) content. Fresh leaves (0.2g) were selected for the evaluation of MDA content by the method of Li et al. (2000). Simply, plant leaves were homogenized in phosphate buffer saline (PBS), then 2 ml of 2-thiobarbituric acid was added. Later, the MDA content was measured at different wavelengths such as 600 nm, 532 nm, and 450 nm respectively. Chlorophyll a, b and carotenoids were determined spectrophotometrically.

### 2.4 Analysis of cell wall composition

Cell wall was isolated by the method of Yang et al. (2011). Lignin was measured at 280 nm wavelength after sample (3mg) acetylated by mixing phenolic hydroxyl with the reaction solution in boiling water (Moreira-Vilar et al., 2014). The plant sample (0.3g) was reacted with ethanol and acetone to obtain a coarse cell wall, and the cell wall substance was dehydrated and subsequently condensed with anthrone under acidic conditions to generate furfural derivatives. The cellulose content was measured by spectrophotometer at 620 nm wavelength. After the plant sample (0.02g) was treated by acid, hemicellulose was converted into reducing sugar, which formed a reddish-brown substance with 3,5-Dinitrosalicylic acid. The absorbance at 540 nm reflected the hemicellulose content. The dry sample (0.1g) reacted with the extract to obtain total pectin, and after reacting with strong acid, its pectin content was measured at 530 nm absorbance.

### 2.5 Collection and determination of organic acids in root exudates

Four plants from each treatment were selected to collect root exudate. Plants were washed with sterilized water and retained in beakers containing 200 mL CaCl_2_ solution (0.5 mM) for 24 h. Plant roots were cleaned with 50 mL of CaCl_2_ solution every 6 hours and water was combined with the preceding 200 mL of CaCl_2_ solution to make a combined 250 mL of root exudate volume. Furthermore, the filtered sample solutions (20 μL) were injected into a 250*4.6 mm reverse phase column with a flow rate of 0.9 mL∙min^-1^ at 35℃ and detected at 210 nm by HPLC to analysis the low molecular weight organic acids (Muhammad et al., 2020).

### 2.6 RNA-seq and gene relative expression analysis

Total RNA was isolated from frozen fresh *S. alfredii* roots by using the TRUE script RT MasterMix (for real time PCR) (DF Biotech., CHENGDU, China) according to the requirements of the instructions (Ge et al., 2021). An aliquot (2 mg) of total RNA was used for first-strand cDNA synthesis using the TransScript First-Strand cDNA Synthesis kit (AiDLAB Biotech, Beijing, P.R. China).

Gene expression level was determined by Quantitative Real-Time PCR Thermal Cycler (Analytik Jena AG, Factory at Konrad-Zuse-Str.1, D-07745 Jena, Germany) with the primer shown in **Table 1** (Zhang et al., 2014). Gene primers were designed according to the sequences obtained from the transcriptome. Results using ACTIN as internal reference, the relative gene expression of each sample and each group was calculated by 2^-△△Ct^. All measurements were conducted in triplicate.

**Table 1:** Sequences of specific-primers used for qRT-PCR.

Gene	Sequence (5’to3’)	TM
*ACTIN*-F	TGTGCTTTCCCTCTATGCC	60
*ACTIN*-R	CGCTCAGCAGTGGTTGTG	
*Nramp3*-F	AAGAAGCAGCTCATGGGTGT	60
*Nramp3*-R	TAAGCTGCGGTGAAGGTTGA	
*Nramp6*-F	TACCTGATAAGACGAGTTGGAA	60
*Nramp6*-R	GCCACCAATATGATCCACAATA	
*HMA2*-F	CTCAAAATGCTGCGAAGCGAAG	60
*HMA2*-R	CTACGCTCTTACAACCATGCCTCG	
*HMA3*-F	CTGTTGCGGACATTGAGA	60
*HMA3*-R	TAGGAGGAGCAGGTTCAG	
*HMA4*-F	GATGCACCAGCACTAGCTAC	60
*HMA4*-R	TCACCAACAAACACGTCCCA	
*CAD*-F	GTACCTGTGTTCTGGTGCTTA	60
*CAD*-R	AGACGAAAGAAATGCTGGAGT	

### 2.7 Statistical analysis

In this study, all the data were presented as the average ± standard deviation of the mean based on the three replicates. The significance of the differences between different treatments was subjected to a One-way ANOVA analysis at *P*<0.05 level by using IBM SPSS 19.0. The figures were made by Origin 2018 and R 4.2.1.

## 3 Results

### 3.1 Exogenous Si promoted the growth and Cd accumulation of *S. alfredii*

**Figure 1:**
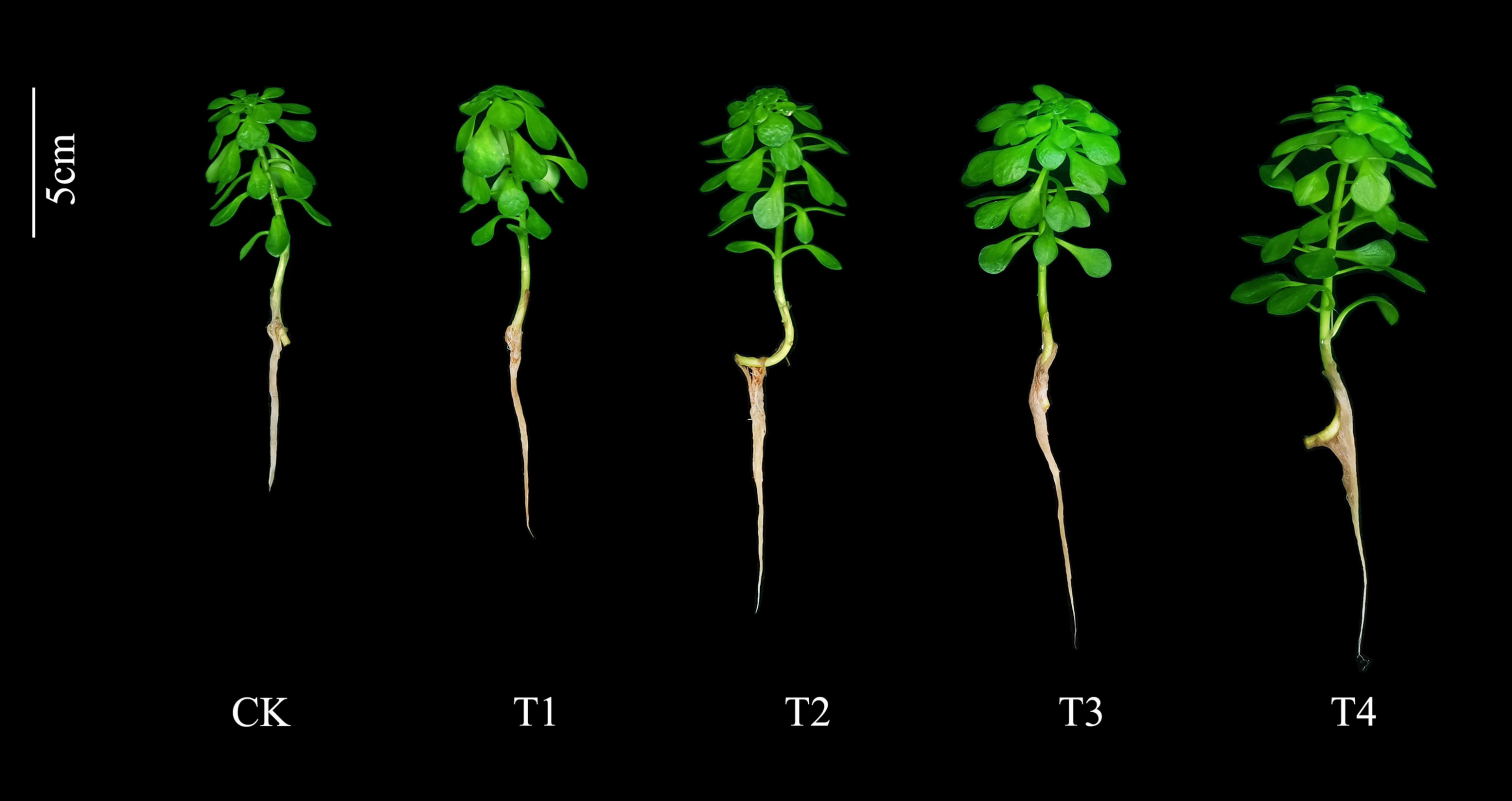
Effects of different Si treatments on the growth of *S. alfredii*. CK: control treatment, T1, T2, T3 and T4 were the Si concentrations of 0.5, 1.0, 1.5 and 2.0 mmol∙L^-1^, respectively.

The data in **Table 2** showed the effects of different Si treatments on the fresh and dry weight of *S. alfredii* after 28 d growth. The fresh and dry weight of shoot and root of *S. alfredii* in Si treatments were significantly increased by 22.67-52%, 21.74-52.17%, 10.71-28.57% and 40-80%, respectively, compared with CK. Moreover, both fresh and dry weights showed the highest value under 2 mM Si treatment. Similarly, Si also significantly (*P*< 0.05) increased the plant height and root length of *S. alfredii* by 10.75-23.26% and 11.90-31.44%, respectively (**Figure 1**).

**Table 2:** Effect of different Si treatments on weight, height and root length of *S. alfredii*.

Treatment	Fresh weight (g∙plant^-1^)		Dry weight (g∙plant^-1^)		Plant height (cm)	Root length (cm)
	shoot	Root	Shoot	Root		
CK	1.50 ± 0.02e	0.28 ± 0.01c	0.23 ± 0.02c	0.05 ± 0.01c	14.23 ± 0.07e	5.63 ± 0.09d
T1	1.84 ± 0.04d	0.31 ± 0.01b	0.28 ± 0.01b	0.07 ± 0.00b	15.76 ± 0.06d	6.30 ± 0.29c
T2	1.89 ± 0.01c	0.32 ± 0.01b	0.29 ± 0.02b	0.08 ± 0.00b	16.02 ± 0.17c	7.05 ± 0.05b
T3	2.18 ± 0.02b	0.34 ± 0.02a	0.35 ± 0.01a	0.09 ± 0.00ab	16.29 ± 0.14b	7.18 ± 0.08ab
T4	2.28 ± 0.03a	0.36 ± 0.01a	0.35 ± 0.01a	0.09 ± 0.01a	17.54 ± 0.14a	7.40 ± 0.10a

CK: control treatment, T1, T2, T3 and T4 were the Si concentrations of 0.5, 1.0, 1.5 and 2.0 mmol∙L^-1^, respectively. Values are mean ± SD and columns denoted with different letters indicate significant difference among treatments at P< 0.05.

**Figure 2:**
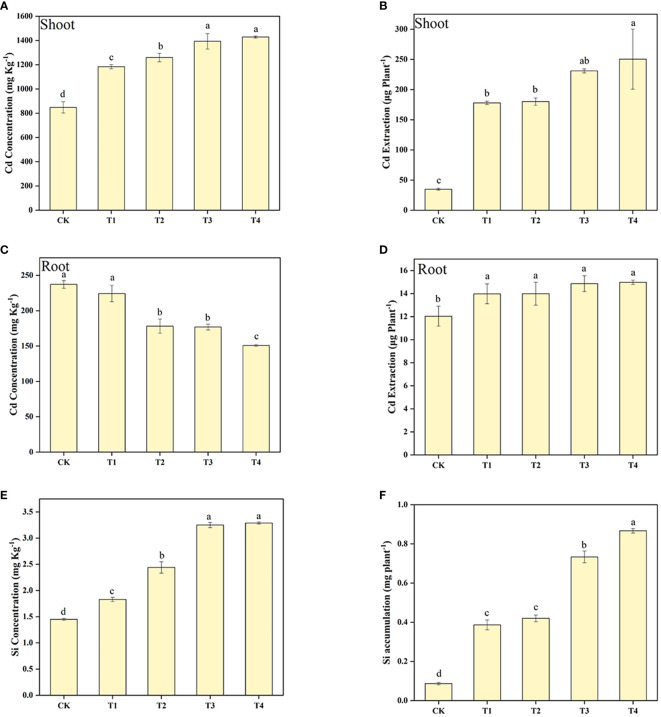
Shoot Cd concentration **(A)**, Shoot Cd accumulation **(B)**, root Cd concentration **(C)**, root Cd accumulation **(D)**, and Si concentration **(E)**, Si accumulation **(F)** of *S. alfredii* under different Si treatments. CK: control treatment, T1, T2, T3 and T4 were the Si concentrations of 0.5, 1.0, 1.5 and 2.0 mmol∙L^-1^, respectively. Values are mean ± SD and columns denoted with different letters indicate significant differences between treatments at *P*< 0.05.

The Cd concentration and extraction in the shoot of *S. alfredii* was enhanced by the increasing of Si levels (**Figure 2**). It can be found that Si treatment significantly (*P*< 0.05) increased the Cd concentration and extraction in the shoot of *S. alfredii* by 39.52-68.44% and 412.39-621.00%, respectively. Moreover, the highest value in concentration and accumulation of Cd in the shoot of *S. alfredii* was found under T4 treatment. Furthermore, exogenous Si significantly (*P*< 0.05) increased Cd extraction in the root of *S. alfredii*, and it increased with higher Si concentrations, but Si showed a negative role on enhancing Cd concentrations in the root of *S. alfredii*. Exogenous Si remarkably increased the Si concentration and accumulation of *S. alfredii* by 25.92-126.61% and 346.02-899.65%, respectively (**Figure 2**). And both the Si concentration and the accumulation displayed the highest values under T4 treatments which was 2.27-fold and 10.00-fold higher than CK treatments.

**Figure 3:**
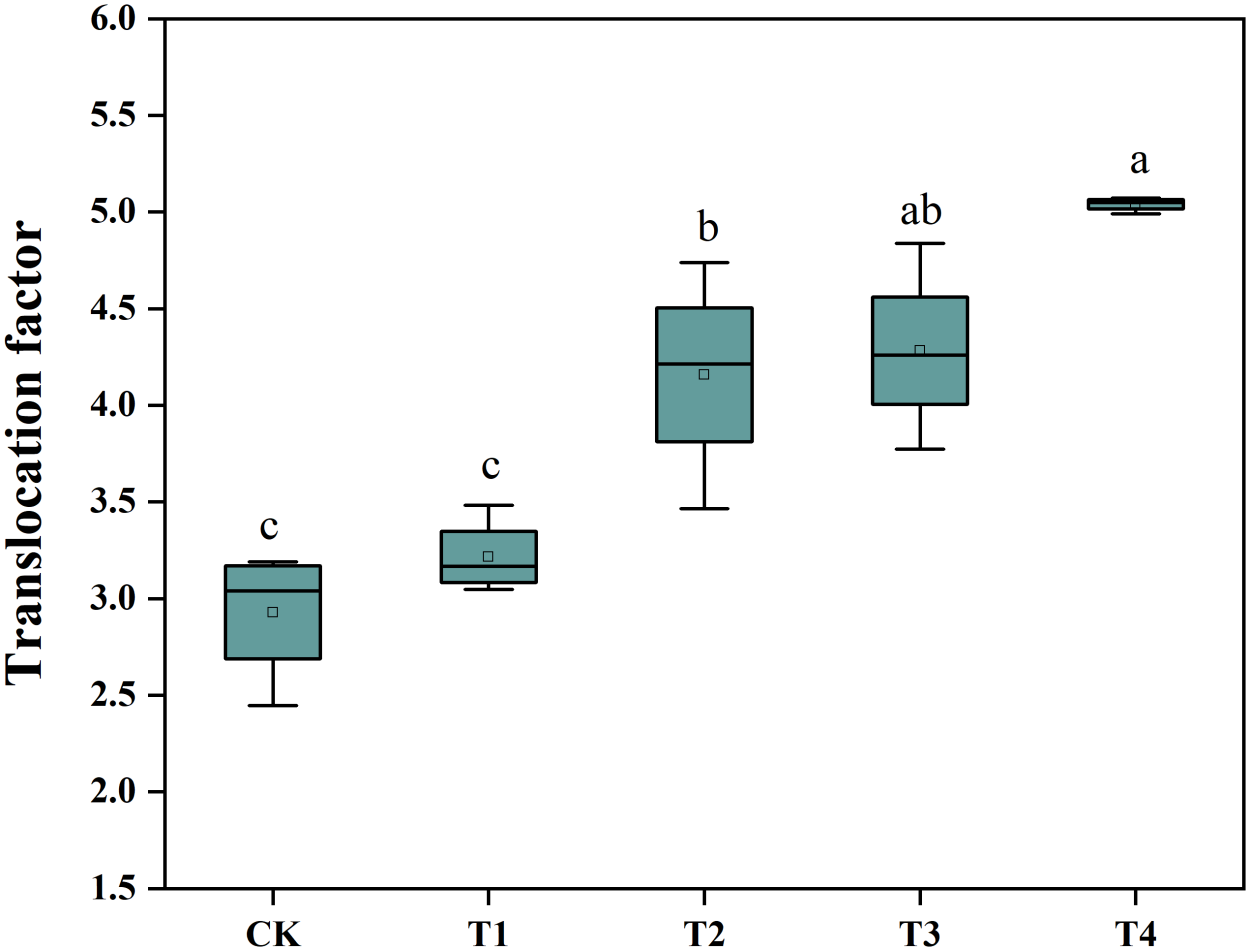
The Cd translocation factor of *S. alfredii*. CK: control treatment, T1, T2, T3 and T4 were the Si concentrations of 0.5, 1.0, 1.5 and 2.0 mmol∙L^-1^, respectively. Values are mean ± SD and columns denoted with different letters indicate significant difference among treatments at *P*< 0.05.

To determine whether Si was able to promote the Cd extraction efficiency of *S. alfredii*, we analyzed the plant Cd translocation factor (TF) (**Figure 3**). The application of Si under the T3 and T4 treatments increased the TF of *S. alfredii* significantly in comparison to the control. The 2 mM Si was effective in improving the TF by 1.72-fold compared to CK. These results indicated that the T4 treatment can significantly (*P*< 0.05) enhance Cd extraction efficiency of *S. alfredii*.

### 3.2 Photosynthetic pigments, lipid peroxidation and antioxidant enzymes activities

**Figure 4:**
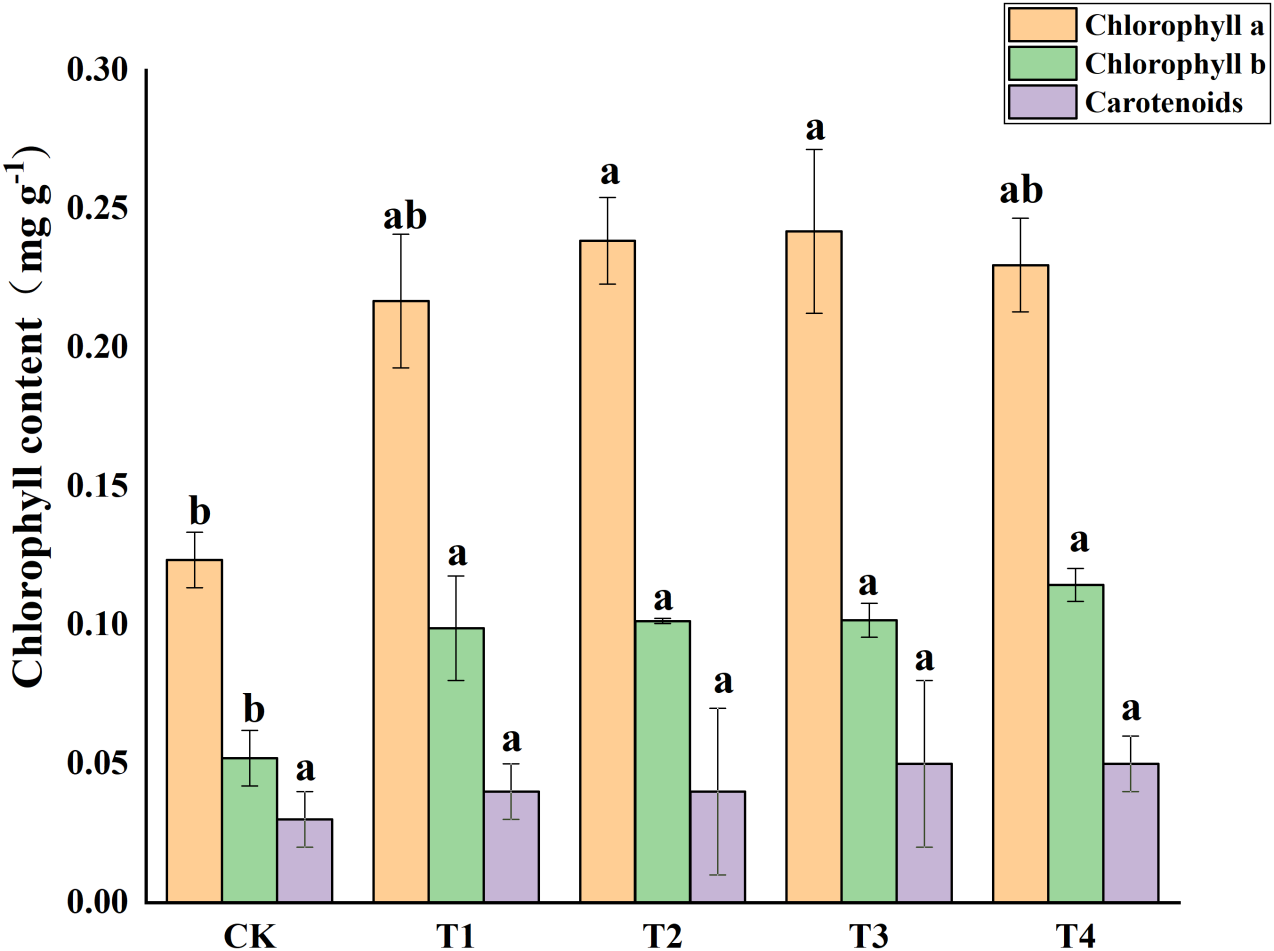
Effects of different Si treatments on chlorophyll content of *S. alfredii*. CK: control treatment, T1, T2, T3 and T4 were the Si concentrations of 0.5, 1.0, 1.5 and 2.0 mmol∙L^-1^, respectively. Values are mean ± SD and columns denoted with different letters indicate significant differences between treatments at *P*< 0.05.

**Figure 5:**
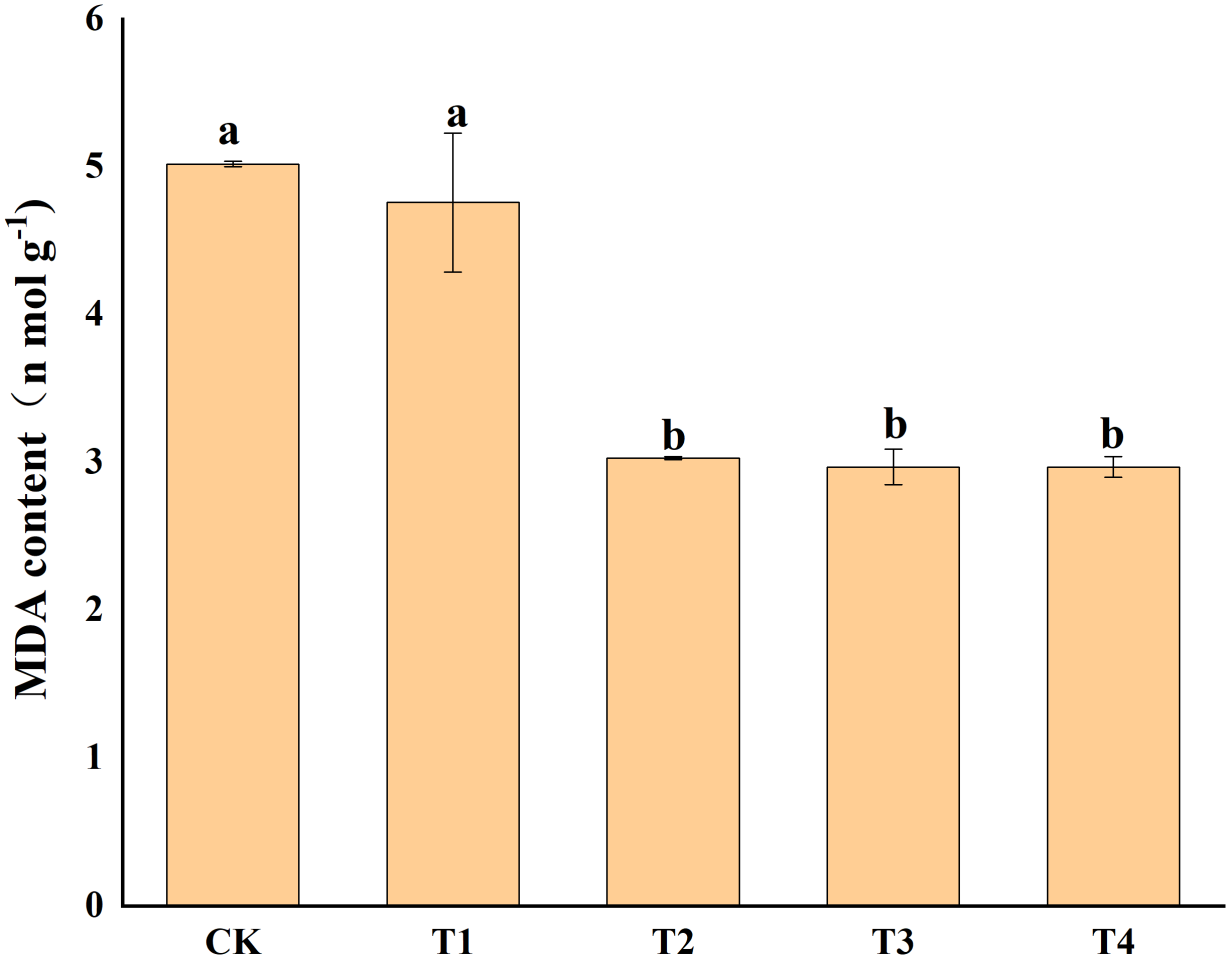
Effects of different Si treatments on MDA content of *S. alfredii*. CK: control treatment, T1, T2, T3 and T4 were the Si concentrations of 0.5, 1.0, 1.5 and 2.0 mmol∙L^-1^, respectively. Values are mean ± SD and columns denoted with different letters indicate significant differences between treatments at *P*< 0.05.

Photosynthetic pigments (chlorophyll a, and b) and other photosynthetic pigments (carotenoids) were analyzed in *S. alfredii* leaves (**Figure 4**). The results showed that exogenous Si reduced Cd toxicity by increasing the total chlorophyll a content and chlorophyll b content by 75.55-95.94% and 90.06-134.04%, respectively. However, exogenous Si had no significant impacts on the carotenoid content of *S. alfredii*. Exogenous Si significantly (*P*< 0.05)decreased the MDA content in Cd stressed plants when compared to the control and the T2, T3 and T4 treatments significantly (*P*< 0.05) decreased the MDA content by 39.62%, 40.94% and 40.94%, respectively (**Figure 5**). These indicated that Si has a positive role on Cd-induced ROS stress.

Antioxidative enzymes play positive impacts in oxidative stress management. The changes of antioxidant enzyme activity in *S. alfredii* under Si treatment were displayed in **Table 3**. The maximum values of POD, SOD and CAT activities were found under T4 treatment. Compared with the control, the T4 treatment significantly (*P*< 0.05) enhanced POD, SOD and CAT activities by 34.92%, 14.57% and 19.63%, respectively. The T1, T2 and T3 treatment also significantly (*P*< 0.05) increased POD, SOD and CAT activities. These results showed that 2mM Si had the best effect in relieving Cd stress of *S. alfredii*.

**Table 3:** Effects of different Si treatments on antioxidant enzymes activities of *S. alfredii*.

	SOD (Ug^-1^)	CAT (Ug^-1^)	POD (Ug^-1^)
CK	143.28 ± 2.57d	515.2 ± 14.92c	1.89 ± 0.06d
T1	145.83 ± 2.00d	523.93 ± 3.19c	1.91 ± 0.02d
T2	150.41 ± 1.21c	531.75 ± 8.86bc	2.08 ± 0.10c
T3	155.54 ± 3.83b	547.89 ± 12.28b	2.36 ± 0.04b
T4	164.16 ± 2.09a	616.84 ± 10.81a	2.55 ± 0.02a

CK, control treatment; T1, T2, T3 and T4 were the Si concentrations of 0.5, 1.0, 1.5 and 2.0 mmol∙L^-1^, respectively. Values are mean ± SD and columns denoted with different letters indicate significant differences between treatments at P< 0.05.

### 3.3 Effects of Si on cell wall components of *S. alfredii*

**Figure 6:**
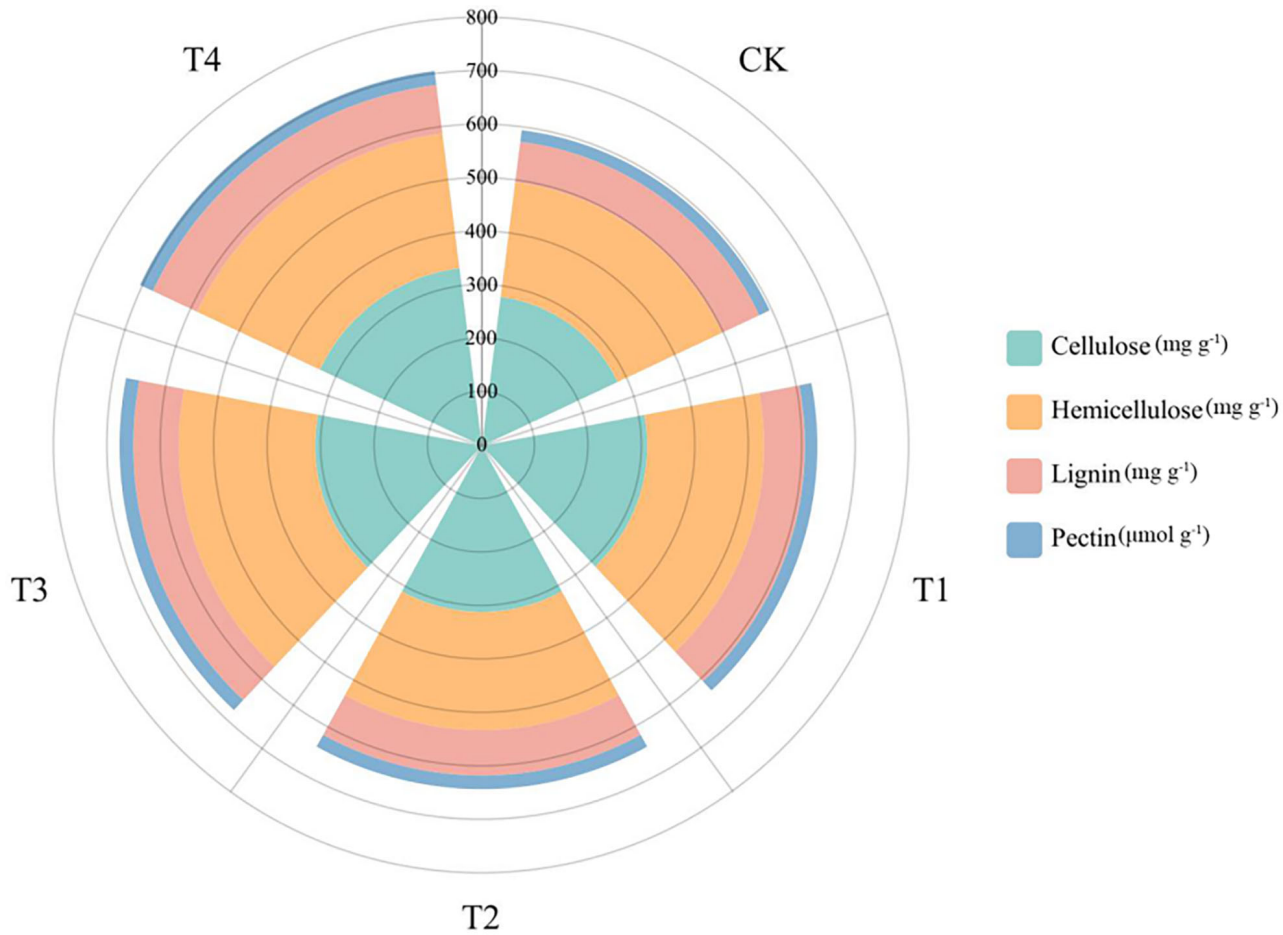
Cell wall components of *S. alfredii* under different Si treatments. CK: control treatment, T1, T2, T3 and T4 were the Si concentrations of 0.5, 1.0, 1.5 and 2.0 mmol∙L^-1^, respectively.

After 28 d growth, the cell wall components of *S. alfredii* were clearly different between Si treatments (**Figure 6**). We found that the cell wall components (pectin, cellulose, lignin, and hemicellulose) and the content of Si in leaves changed greatly. For instance, compared to the CK, Si significantly increased the lignin content, cellulose content, hemicellulose content and pectin content of *S. alfredii* by 0.41-20.43%, 10.37-19.39%, 0.73-17.70% and 8.15-23.30%, respectively.

### 3.4 Organic acids in root exudates of *S. alfredii* influenced by Si treatments

**Figure 7:**
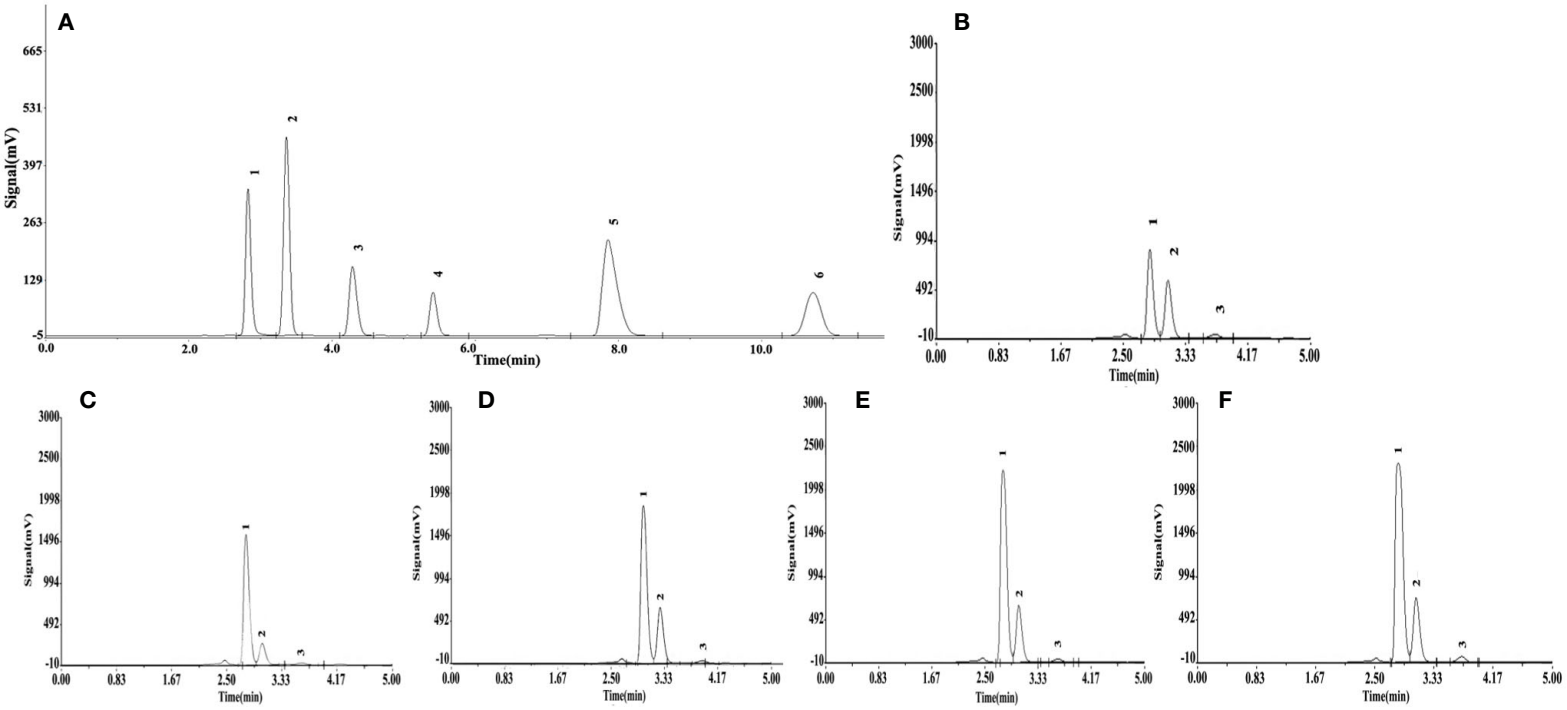
Effects of different Si treatments on organic acids content in root exudates of *S. alfredii*. Organic acid standard solutions **(A)**, root exudates of *S. alfredii* under different Si treatments (B~F). **(B)**: control treatment, **(C, D, E, F)** were the Si concentrations of 0.5, 1.0, 1.5 and 2.0 mmol∙L^-1^, respectively.

Significant differences (*P*< 0.05) in organic acid concentrations were found under different Si treatments (**Table 4**). The organic acid standard solutions are oxalic acid, tartaric acid, L-malic acid, acetic acid, citric acid and D-malic acid in the peak order in **Figure 7**. Tartaric acid, oxalic acid and L-malic acid were detected in the root exudates of *S. alfredii* in this study. The different Si application resulted in significant increases in excretion of root exudates by *S. alfredii* (**Figure 7**). The maximum values for tartaric acid, oxalic acid and L-malic acid were all found under 2.0 mM Si. The T4 treatment significantly (*P*< 0.05) promoted the excreted concentrations of oxalic acid, tartaric acid and L-malic acid by 114.78%, 136.83% and 203.08%, respectively, compared with CK (**Table 4**).

**Table 4:** Effects of different Si treatments on the organic acids concentrations in root exudates of *S. alfredii*.

	oxalic acid (mg∙L^-1^h^-1^)	tartaric acid (mg∙L^-1^h^-1^)	L- malic acid (mg∙L^-1^h^-1^)
CK	2.03 ± 0.03e	5.05 ± 0.04d	0.65 ± 0.01e
T1	2.13 ± 0.06d	4.66 ± 0.44d	0.76 ± 0.02d
T2	2.49 ± 0.06c	8.93 ± 0.08c	1.57 ± 0.03c
T3	3.29 ± 0.01b	9.75 ± 0.02b	1.75 ± 0.04b
T4	4.36 ± 0.03a	11.96 ± 0.15a	1.97 ± 0.03a

CK, control treatment; T1, T2, T3 and T4 were the Si concentrations of 0.5, 1.0, 1.5 and 2.0 mmol∙L^-1^, respectively. Values are mean ± SD and columns denoted with different letters indicate significant differences between treatments at P< 0.05.

### 3.5 Gene expression of *S. alfredii* under different Si treatments

**Figure 8:**
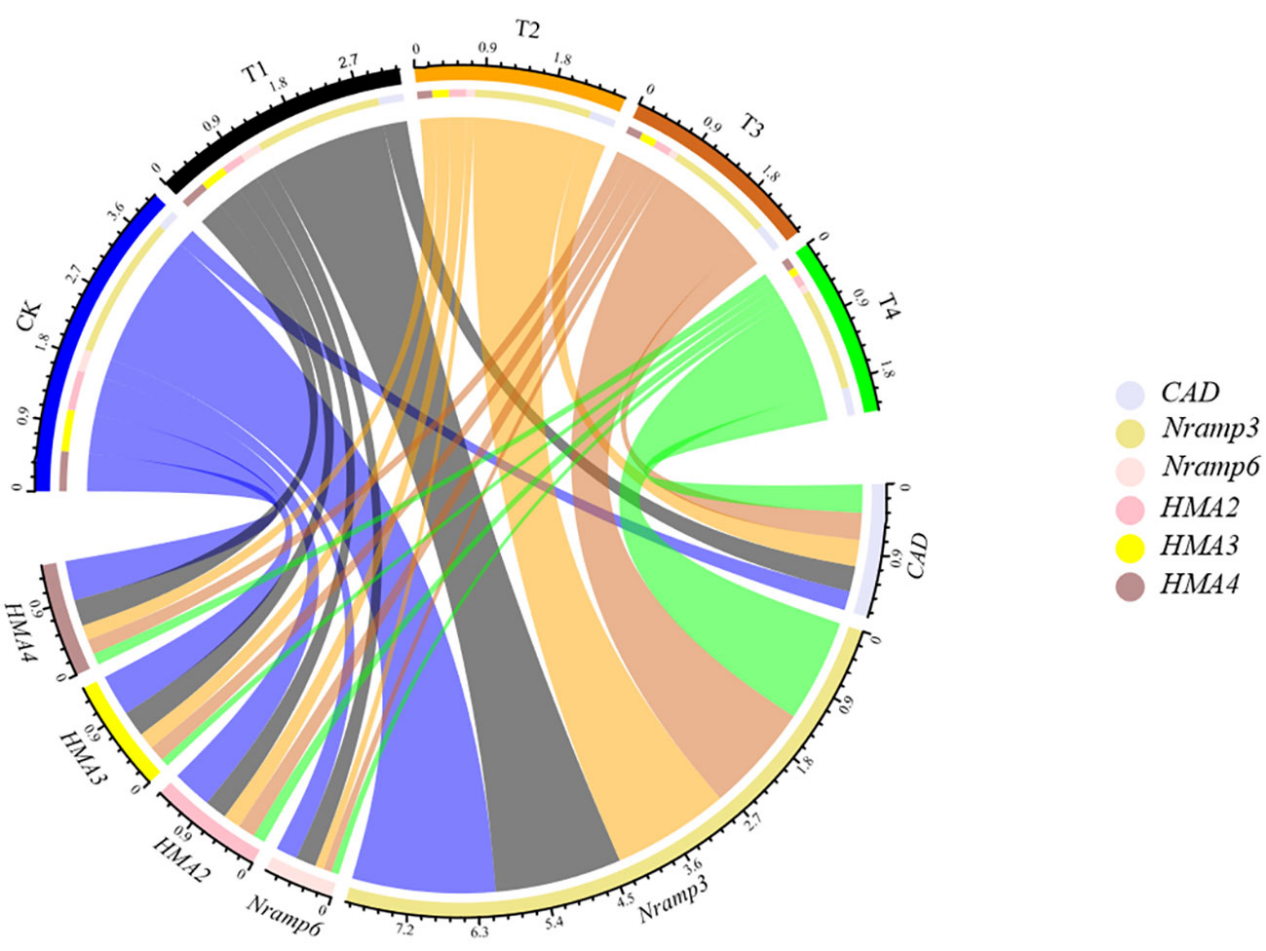
The expression level of related genes (*SaNramp3*, *SaNramp6*, *SaHMA2*, *SaHMA3*, *SaHMA4* and *SaCAD*) in the root of *S. alfredii* under different Si treatments. CK: control treatment, T1, T2, T3 and T4 were the Si concentrations of 0.5, 1.0, 1.5 and 2.0 mmol∙L^-1^, respectively.

To test whether Si could regulate the symplastic transport routine of Cd, the expression of genes, including *SaNramp3*, *SaNramp6*, *SaHMA2*, *SaHMA3*, *SaHMA4* and *SaCAD*, potentially involved in *S. alfredii* Cd transport and cell wall synthesis was investigated (**Figure 8**). After 28d growth, the gene expression profile of *S. alfredii* was clearly different between treatments (**Figure 8**). Compared to the control, the Si application significantly down-regulated the expression of *SaNramp3*, *SaNramp6*, *SaHMA3*, *SaHMA2* and *SaHMA4* by 11.46-28.23%, 6.61-65.19%, 38.47-80.87%, 44.80-69.85% and 33.96-71.70% in the root of *S. alfredii*, respectively. While the Si treatments significantly increased the *SaCAD* expression, and the highest level was found under T4 treatment which was 1.46-fold higher than the CK.

## 4 Discussion

### 4.1 Influences of Si on growth and Cd accumulation of *S. alfredii*

This study aims to explore the feasibility of Si in enhancing the efficiency of phytoextraction by *S. alfredii*. According to the results obtained here, exogenous Si application significantly promoted Cd accumulation by *S.alfredii*, indicating Si enhanced the efficiency of phytoextraction. The success of phytoextraction depends largely on the plant biomass as well as the metal concentrations in plants (Eissa, 2017). Our results showed that Si promoted growth of *S. alfredii* and significantly enhanced the biomass of *S. alfredii* (**Table 2**). On the one hand, Si might improve mineral nutrition by reducing Cd toxicity of *S. alfredii* (Mukarram et al., 2022), and Si was shown to accumulate in the leaf apoplast as polymer and thereby act as a defense line preventing Cd from entering cells (Silva et al., 2017; Rahman et al., 2021). In addition, Si has been shown to alleviate the lipid peroxidation of membranes and promote the synthesis of chlorophyll, thus enhancing photosynthesis that is ultimately responsible for plant biomass production (Rastogi et al., 2021). On the other hand, Si altered the biosynthesis, structure, and function of the cell wall by regulating the production of lignin, pectin, cellulose and hemicellulose, and subsequently promote the absorption of nutrients by *S. alfredii* (Sheng and Chen, 2020). It is also possible to prevent plant root cells from being poisoned by Cd by increasing transcription of Cd transport genes (**Figure 8**), thus promote the development of roots, and then increase the absorption of nutrients, which is beneficial to the growth of plants (Ma and Yamaji, 2006; Ma and Yamaji, 2015).

Another important factor that determines phytoextraction efficiency is the metal concentration in the hyperaccumulators. In this study, Si significantly increased Cd concentrations of *S. alfredii* (**Figure 2**). Several reasons may contribute to this. Firstly, the increased organic acids excretion, induced by Si application, contributes to the uptake and accumulation of Cd by *S. alfredii* (Tao et al., 2019). Previous studies had shown that oxalic acid, malic acid and tartaric acid all has the ability to make Cd complexes, and participate in various physiological metabolic processes in plants, including the uptake, transport, storage and detoxification of heavy metals (Ma et al., 2001). They have been shown to contribute to Cd accumulation in *S. alfredii* probably by releasing Cd and simultaneously complexing Cd, thus improving the Cd phytoextraction efficiency by *S. alfredii* (Tao et al., 2019). Besides, hyperaccumulators were reported to activate and chelate heavy metals by secreting a series of chelators such as low molecular weight organic acids (Qiao et al., 2022). In agreement with previous results, an increase in organic acids is beneficial for the transportation of Cd in the shoots of *S. alfredii*. Which was supported by the increasing of the TF (**Figure 3**). Secondly, the expression of Cd transporter gene was down-regulated by exogenous Si application in the root of plant, which was also beneficial for accumulation of Cd in the shoot (**Figure 8**). In summary, our study demonstrated that the presence of Si increased biomass and Cd concentrations in *S. alfredii*, which helps in enhancing Cd extraction efficiency.

### 4.2 Exogenous Si alleviated the Cd toxicity of *S. alfredii*

One of the most important mechanisms that hyperaccumulators reduce Cd damage is by improving photosynthesis and maintaining membrane stability. Photoreaction site is one of the most sensitive sites to heavy metal stress (Ashfaque et al., 2017; Bayçu et al., 2018; Rusinowski et al., 2019). Previous studies have confirmed that heavy metals could inhibit the photosynthetic pigments of plants (Araújo et al., 2017; Haque et al., 2021). In our study, Si increased the chlorophyll a and b content of *S. alfredii* (**Figure 4**). A possible explanation may be that Si is able to modulate expression of photosynthetic genes or promote plants to absorb more mineral nutrients, thus prevent damage to chloroplast membrane and thylakoids under metal stress (Feng et al., 2010; Song et al., 2014; Jia et al., 2021; Zhao et al., 2022). In addition, Si promoted the uptake of nutrients involved in photosynthetic process of plants (Rengel, 1999), which may also explain our results. Moreover, Si application has been shown to decrease ROS production or increase enzymatic antioxidant activity which ultimately reduce oxidative stress, thus increasing the chlorophyll contents (Kim et al., 2017). MDA is an indicator to measure membrane lipid peroxidation, and its level can reveal the damage degree of membrane structure and function (Gheshlaghpour et al., 2021). Previous studies have suggested that Cd stress can lead to the accumulation of MDA in plants (Muhammad et al., 2020; Pandian et al., 2020; Yuan et al., 2023). In our study, exogenous Si significantly minimized the content of MDA in *S. alfredii* (**Figure 5**). These results indicate that Si helps to eliminate various ROS species caused by Cd stress, thus ensuring the cell membranes not damaged. Similarly, Farooq et al. (2013) also confirmed that Si treatment helped to maintain the normalization of cell membrane.

Toxicity of Cd is also usually manifested by overproduction of toxic ROS (Vaculík et al., 2021). Previous research reported that excessive ROS accumulated in plants under Cd stress was able to lead to plant growth retardation (Zhou and Qiu, 2005), inhibition of photosynthetic pigment synthesis (Wahid et al., 2008; Farooq et al., 2013), cell wall looseness during cell expansion (Berni et al., 2019), and enzyme inactivation (Farooq et al., 2013). The antioxidant enzymes (POD, CAT and SOD) are considered as the first barrier against ROS (Zhang et al., 2009; Gong et al., 2019; Zulfiqar and Ashraf, 2022). Improving antioxidant enzyme activity is an important way to support hyperaccumulator to alleviate metal toxicity (Asghari et al., 2020). Numerous studies have confirmed that the activity of antioxidant enzymes was increased due to Cd toxicity (Ahmad et al., 2016; Huang et al., 2019; Rahman et al., 2020; Zhou et al., 2022). In this study, it was found that Si application significantly increased the activity of antioxidant enzymes (**Table 3**). Ranjan et al. (2021) and Bhat et al. (2019) also reported that the relief of metal toxicity by Si was mainly due to a significant increase in enzymatic antioxidants. Our results demonstrated that Si might protect *S. alfredii* under Cd stress by enhancing the activity of defense enzymes.

Cell wall deposition is an important mechanism for plants to alleviate Cd toxicity. Research showed that Cd in deposited in the cell wall accounts for the majority of Cd in plants under high Cd stress (Yu et al., 2023). And Si has also been shown to protect cells of the plant against heavy metals toxicity by increasing the thickness and strength of the cell wall (Gall et al., 2015). Therefore, the tolerance and mitigation of Cd in *S. alfredii* might largely depend on cell wall deposition. The fixation of Cd by the plant cell wall was shown to be mainly dependent on various compounds displaying electronegative coordination provided by macromolecules such as cellulose, hemicellulose, lignin and pectin (Fu et al., 2011; Liu et al., 2014; Zhao et al., 2019; Wei et al., 2021; Yu et al., 2023). Chemically, the cell wall is mainly composed of three layers with structural components including pectin, cellulose, lignin and hemicellulose (Sajad et al., 2020). It is reported that Cd has negative effects on biosynthesis of pectin, lignin, cellulose and hemicellulose (Loix et al., 2017; Luyckx et al., 2021). In this study, we found that Si application remarkably increased the amount of lignin, pectin, cellulose and hemicellulose in *S. alfredii* (**Figure 6**), which were positively correlated to the increasing Si concentrations in plants (**Figure 2**). This was agreement with previous study which demonstrated that the content of lignin, pectin, cellulose and hemicellulose in plants were positively correlated to the content of Si (Zhang et al., 2015). There is evidence that Si enhances the antioxidant defense system of plants, alleviates the damage of ROS to cell wall, and promotes the synthesis of cell wall components (Xiong et al., 2010), such as the influence of POD activity on lignin biosynthesis and metabolism (Liu et al., 2019). Moreover, numerous studies have confirmed that the increase in cell wall components related to the gene expression, protein synthesis and transcription that caused by Si (Shafi et al., 2015; Haddad et al., 2019; Ranjan et al., 2021). Therefore, the influence of Si on the cell wall components is one of the important internal mechanisms for Si to reduce Cd toxicity of *S. alfredii*.

Another important mechanisms of Si in relieving Cd toxicity of *S. alfredii* is due to stimulating production and excretion of organic acid exudate from plants to subsequently chelate Cd^2+^ (Bhat et al., 2019). Tao et al. (2019) reported that oxalic acid, malic acid and tartaric acid are the main organic acids secreted by *S. alfredii*, which was similar to our results (**Figure 7**). One possible explanation may be that under Cd stress, exogenous Si reduced the production of excessive ROS, improved the photosynthetic efficiency, and then enhanced the secretion of organic acids in plant roots (Muhammad et al., 2020). Furthermore, organic acids are an middle step of photosynthesis through the tricarboxylic acid cycle (TCA) of the plant, thus, they may be involved with many physiological processes (Ubeynarayana et al., 2021). And previous studies showed that the contents of malic acid, tartaric acid and oxalic acid secreted by the roots of *S. alfredii* under Cd stress were increased (Chen et al., 2014; Muhammad et al., 2020), which may be a spontaneous adaptative mechanism of *S. alfredii* under Cd stress (Muhammad et al., 2020). The metal-organic acid anions-complex formed from this interaction reduced the toxicity of Cd to plants (Barcelo et al., 1993; Tanwir et al., 2015; Fan et al., 2016). In our study, exogenous Si promoted an increase in malic acid, tartaric acid and oxalic acid secreted by roots (**Table 4**). Therefore, we speculate that Si helps to activate various enzymes in the TCA cycle of plants, thus increasing the production of organic acids secreted by the roots and then alleviate the Cd toxicity (Rengel, 2002).

### 4.3 Root genes regulated tolerance and transportation of Cd were affected by Si

The expression levels of selected genes that regulate tolerance and transportation of Cd in roots of *S. alfredii* were analyzed. *SaNramp3* was reported to play an essential role in conferring tolerance and accumulation of Cd (Feng et al., 2018). *SaNramp6* was a Cd transporter located in the vesicular-shaped endomembrane compartment (Cailliatte et al., 2009). *HMA3* is a transport gene located in vacuole membrane, and participates in metal detoxification by isolating Cd into the vacuole (Chaudhary et al., 2016). *HMA2* and *HMA4* are proteins, which were shown to help excreting Cd^2+^ (Wu et al., 2015). The cinnamyl alcohol dehydrogenase of *S. alfredii* (*SaCAD*) is an essential enzyme in regulating lignin biosynthesis, which plays an critical role in enhancing Cd fixation on the cell wall (Qiu et al., 2018). It was reported that one of the strategies of *S. alfredii* in alleviating heavy metal stress in the root systems was expression of *SaNramp3* and *SaHMA3*, with their gene products being able to transport heavy metals to the vacuoles and subsequently store them in vacuoles (Yang et al., 2022). In this study, the expressions of *SaNramp3* and *SaHMA3* were down-regulated in the root of *S. alfredii* (**Figure 8**), which indicated that other mechanisms contributing more essentially and importantly than vacuolar sequestration might evolve (Peng et al., 2017). Besides, we found that the expression of *SaCAD* involved in plant lignin synthesis was significantly increased (**Figure 8**). The overexpression of *SaCAD* changed the cell wall composition of *S. alfredii* and might induce more Cd deposition in *S. alfredii* cell wall. In consistent with our results, Lu et al. (2020) also confirmed that *SaCAD*-overexpressing plants were capable of retaining more metal in the cell wall. Similarly, the down-regulation of *SaHMA2*, *SaHMA4* and *SaNramp6* will also discharge Cd from roots and accumulation of Cd in roots, so that the roots have a better environment for growth, which may help reduce the toxicity of Cd to root cells of plant and promote *S. alfredii* growth (Hanikenne et al., 2008; Chen et al., 2016; Zhang et al., 2016).

The down-regulation of genes encoding heavy metal transporters was not only alleviate the Cd stress of plants, but also explain the increase of Cd concentrations in the shoot of *S.alfredii*. *SaNramp3*, *SaHMA3* and *SaHMA2* are considered to be important transporters promoting root-to-shoot translocation of *S.alfredii*. Since the root-to-shoot translocation of Cd has been shown to be controlled by processes such as xylem loading and vacuolar sequestration, the down-regulation of *SaNramp3*, *SaHMA3* and *SaHMA2* indicated that the vacuole-isolated metal in the root of *S. alfredii* decreased, which resulting in an increased flux of metals towards the xylem, thus contribute to an increased transportation and accumulation of Cd in shoots (Mendoza-Cózatl et al., 2011; Ueno et al., 2011; Takahashi et al., 2012; Wu et al., 2015; Lu et al., 2020; Ge et al., 2021). Thus, the low expression of *SaNramp3*, *SaHMA3* and *SaHMA2* in roots could contribute to the high Cd accumulation in the shoot of *S. alfredii* (**Figure 8**). Our results indicates that the change in transport gene expression mediated by Si is beneficial to alleviate the growth inhibition of *S. alfredii* under Cd stress and increase the Cd accumulation by *S. alfredii*.

## 5 Conclusions

Our study revealed that exogenous Si application (mainly at 2 mM level) significantly promoted the biomass, Cd translocation factor and concentration in the shoots of *S. alfredii*, which led to a significantly increase in the Cd accumulation. In addition, this study suggested that Si application alleviated Cd toxicity of *S. alfredii* by: (i) increasing chlorophyll contents, (ii) improving antioxidant enzyme and lipid peroxidation of membrane, (iii) enhancing cell wall components, (iv) raising the secretion of low molecular weight organic acids. Moreover, the regulation of genes that involved in Cd detoxification such as *SaNramp3*, *SaNramp6* and *SaCAD* may also involve in the enhanced Cd resistance of *S. alfredii* after Si application. Our results demonstrated that Si facilitated Cd accumulation by *S. alfredii*. However, future research needs to corroborate the results of the present work in field phytoextraction trial.

## Data availability statement

The original contributions presented in the study are included in the article/supplementary material. The raw sequence data in this study have been deposited in the NCBI database (https://www.ncbi.nlm.nih.gov/sra/) with the dataset accession number: SRR23460268. Further inquiries can be directed to the corresponding authors.

## Author contributions

YH: Investigation, Methodology, Writing-original draft. XZ: Formal analysis, Investigation, Data curation. AS: Methodology, Formal analysis. YY: Data curation, Software. CR: Writing – review & editing. TZ: Conceptualization, Resources, Writing – review & editing. SX: Conceptualization. WY: Supervision, Project administration, Funding acquisition, Writing – review & editing. All authors contributed to the article and approved the submitted version.
